# Extracorporeal carbon dioxide removal (ECCO2R) in COPD and ARDS patients with severe hypercapnic respiratory failure. A retrospective case-control study

**DOI:** 10.3906/sag-2012-151

**Published:** 2021-08-30

**Authors:** Volkan İNAL, Serdar EFE

**Affiliations:** 1 Division of Critical Care, Department of Internal Medicine, Faculty of Medicine, Trakya University, Edirne Turkey

**Keywords:** Extracorporeal carbon dioxide removal, ECCO2R, hypercapnic respiratory failure, artificial membranes, survival, intensive care

## Abstract

**Background/aim:**

Treatment of severe hypercapnic respiratory failure (HRF) has some challenges in patients with chronic obstructive pulmonary disease (COPD) and acute respiratory distress syndrome (ARDS), especially when lung protective ventilation (LPV) strategies are required. Extracorporeal CO_2_ removal (ECCO2R) therapy is an emerging option to manage hypercapnia while allowing LPV in these cases. However, further data on ECCO2R use is still needed to make clear recommendations.

**Materials and methods:**

This study was conducted on patients admitted to intensive care unit (ICU) between January 1st, 2016 to December 31st, 2019. The medical records were retrospectively scanned in institutional software database. Patients who received invasive mechanic ventilation (iMV) support due to severe HRF related to COPD or ARDS were included in the analyses. Patients were grouped according to treatment approaches as that ECCO2R therapy in addition to conventional treatments and conventional treatments alone (controls). Groups were compared for 28-day survival, iMV duration, and length of stay (LOS).

**Results:**

ECCO2R therapy was noted in 75 of the cases among included 395 patients (COPD n = 256, ARDS n = 139) out of scanned 1715 medical records. The survival rate of ECCO2R patients was 68% and significantly higher than 58% survival rate of controls (p = 0.025), with relative risk reduction (RRR) = 0.16, absolute risk reduction (ARR)= 0.10, number need to treat (NNT) = 10, and odds ratio (OR) = 1.5. In addition, iMV duration (12.8 ± 2.6 vs. 17.1 ± 4.9 days, p = 0.007) and LOS (16.9 ± 4.1 vs. 18.9 ± 5.5 days, p = 0.032) were significantly shorter than controls. Repeated measure analyses showed that LPV settings were successfully provided by 72 h of ECCO2R therapy. Subgroup analyses according to diagnoses of COPD and ARDS also favored ECCO2R.

**Conclusion:**

ECCO2R therapy significantly improved survival, iMV duration and LOS in patients with severe HRF due to COPD or ARDS, and successfully provided LPV approaches. Further studies are needed to assess promising benefits of ECCO2R therapy.

## 1. Introduction

Hypercapnic respiratory failure is defined as a condition that plasma pH < 7.35 and partial arterial carbon dioxide (CO_2_) pressure (PaCO_2_) > 49 mmHg [1]. In more severe cases (pH < 7.25 and PCO_2_ > 60 mmHg), when persisted against attempts of medical therapy and noninvasive ventilation, invasive mechanical ventilation (iMV) support and intensive care unit (ICU) admission is required [1,2]. Severe hypercapnia, whether associated with chronic obstructive pulmonary disease (COPD) or acute respiratory distress syndrome (ARDS), is an independent risk factor for patient mortality [3]. In addition, iMV support, used in treatment of severe hypercapnia, has its own consequences such as, ventilatory associated pneumonia, ventilator-induced lung injury (VILI), extubation failure, prolonged intubation, and iMV dependence. In recent decades, the lung protective ventilation (LPV) concept (low tidal volume and pressure-limited ventilation) has been presented with more favorable outcomes than traditional ventilation approaches in patients with respiratory failure [4]. LPV has been reported to decreased VILI, facilitated extubation, and improved clinical outcomes [5].

However, LPV has an undesired consequence, progressing hypercapnia [5]. In order to break this deadly vicious cycle, some clinicians have proposed removing excessive CO_2_ by an adjunct extracorporeal device. It was a former concept, first introduced by Kobolow and Gattinoni almost 40 years ago and has re-gained attention [6–8]. This extracorporeal carbon dioxide removal (ECCO2R) technic can be described as that CO_2_ is removed by an attached artificial lung while oxygen delivered through the natural lung. Results of preliminary studies on accountability of ECCO2R therapy have exceeded the clinical expectations [2,9–14]. However, ECCO2R has been a rescue treatment option in patients with severe hypercapnic respiratory failure until recent years [13–16].

The later technological improvements and experiences in ECCO2R have provided promising clinical outcomes with lower adverse event rates [11–14,17]. Finally, ECCO2R therapy has been recommended in the treatment of ARDS and acute exacerbations of COPD to apply LPV while managing hypercapnia, and to achieve targets of pH > 7.30, respiratory rate (RR) < 20–25 breaths/min., driving pressure (ΔP) < 14 cm H_2_O and plateau pressure (Pplat) < 25 cm H_2_O, by the European Consensus Report [18]. Some prospective feasibility studies have still been recruiting cases (i.e., the REXECOR trial - NCT02965079, the REST trial - NCT02654327). However, further feasibility studies and more evidence-based data are required to make stronger recommendations.

ECCO2R has been successfully used in treatment of patients with severe HRF for about 5 years in our ICU. We conducted a retrospective data analyze of our patients who were received ECCO2R therapy. The objective was to elucidate and document favorable effects of ECCO2R therapy against conventional treatments alone at respect of 28-day survival, iMV duration, and ICU length-of-stay (LOS).

## 2. Materials and methods

This retrospective study was conducted in Trakya University Training and Research Hospital, in Turkey, between October 5th–November 27th, 2020. The approval was received from Bioethical Board of Trakya University (no. = 2020/199–09/05). Informed consent for “processing and publishing personal medical data for scientific purposes” has been obtained at ICU admissions (institutional policy) from patients or legally authorized surrogates when patients were intubated, ventilated, unconscious or sedated.

### 2.1. Case definitions

The case definitions were based on those “2016 British Thoracic Society/Intensive Care Society (BTS/ICS) Guideline for the ventilatory management of acute hypercapnic respiratory failure in adults” for hypercapnic respiratory failure, “2019 The Global Initiative for Chronic Obstructive Lung Disease (GOLD)” for COPD and “2012 The Berlin Definition” for ARDS [1,19,20].

### 2.2. ECCO2R indications

According to practice instructions of our clinic, ECCO2R therapy has been applied in patients who met all requirements of these five-criteria; (1) persisting severe hypercapnic respiratory acidosis (pH < 7.15) despite optimized attempts of iMV for more than 3 h, (2) lung protective ventilation was required but hypercapnia was undesirable or contraindicated, (3) no contraindications for canulation and systemic anticoagulation, (4) hemodynamic status was manageable, (5) the underlying disease was reversable or no markers of poor short-term prognosis.

### 2.3. ECCO2R procedure

The veno-venous decap system (Hemodec, Salerno, Italy) with a small membrane lung (0.3 to 1.35 m^2^) connected in series with a roller pump and low flow rates (< 500 mL/min) was used for ECCO2R therapy [5,21,22]. Vascular accesses were provided by percutaneous inserted double-lumen catheters into internal jugular or femoral vein. Unfractionated heparin continuous infusion protocol was used according to activated partial thromboplastin time readings as recommended.

### 2.4. Patients’ selection

The medical records of patients admitted to ICU between January 1st, 2016 to December 31st, 2019 were retrospectively scanned in institutional software database. Diagnoses were searched by ICD-10 codes (J96, J44, J80). All patients who required iMV support due to hypercapnic respiratory failure related to COPD or ARDS diagnoses were included. Patients detected with both of COPD and ARDS diagnoses were excluded to avoid case-mix bias in subgroup comparisons.

### 2.5. Comparisons

**Figure 1:**
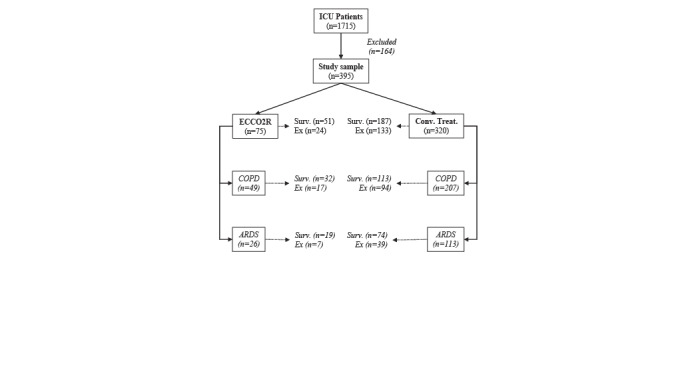
Study diagram representing allocations and comparisons. ICU; intensive care unit, ECCO2R; extracorporeal CO2 removal, COPD; chronic obstructive pulmonary disease, ARDS; acute respiratory distress syndrome, conv. treat.; conventional treatment alone, surv.; survival.

Patients were grouped according to treatment approaches as they were received ECCO2R therapy in addition to conventional treatments (cases), and conventional treatments alone and not received ECCO2R (controls). Case and control groups were compared for 28-day survival, iMV duration, and ICU LOS (see Figure 1). The changes in clinical and laboratory parameters after 72 h of procedure were also analyzed.

**Figure 2:**
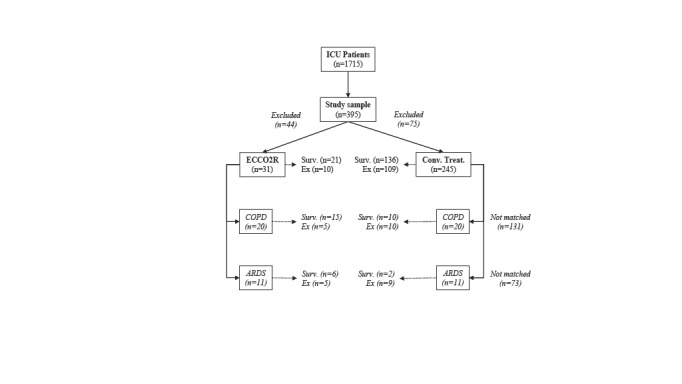
Diagram representing secondary re-test procedure. Additional exclusions and best 1:1 matching process. ICU; intensive care unit, ECCO2R; extracorporeal CO2 removal, COPD; chronic obstructive pulmonary disease, ARDS; acute respiratory distress syndrome, conv. treat.; conventional treatment alone, surv.; survival.

Finally, a strict 1:1 matching was processed to gain a precise aspect and to re-test hypothesis. Additional exclusion criteria were re-defined as; patients with age < 18 and 90 <, obstetrics, hematologic and oncologic diagnoses, acute decompensated heart failure, acute coronary syndromes, profound distributive shock, and ECCO2R therapy less than 72 h. To assure a better comparability, ECCO2R patients were best 1:1 matched with controls by sequential organ failure assessment (SOFA) scores corresponding to their disease severity status. Unmatched control patients were excluded, while we downsized the sample. Resulting COPD_ECCO2R_: COPD_Control _and ARDS_ECCO2R_: ARDS_Control_ subgroups were within compared for survival. This matching process was presented in Figure 2.

The operators were blinded for outcomes throughout selection, allocation, matching, and exclusion procedures, provided by software concealment.

### 2.6. Data collection

Patients’ sex, age, SOFA scores, total iMV duration, ICU LOS, and 28-day survival status were collected. In order to evaluate improvements by ECCO2R procedure; arterial blood gas (ABG) parameters (pH, PaCO_2_, PaO_2_) and ventilatory parameters (P/F ratio: PaO_2_/FiO_2_ ratio, PEEP; positive end expiratory pressure, Pplat, ΔP[driving pressure] = Pplat - PEEP, Tv/PBW; tidal volume/predicted body weight) were noted at the initiation (t = 0) and at the 72nd hours of therapy (t = 72). Clinical (RR; respiratory rate, HR; heart rate, MAP; mean arterial pressure) and laboratory parameters (hemoglobin, PLT, PT-INR, aPTT-ratio) were also collected to detect any probable deteriorations. Total ECCO2R duration, the mean pump flow and sweep gas flow rates were recorded.

Any severe adverse effect related to ECCO2R procedure (worsening hypoxemia, hemolysis, anti-coagulation or canula related bleeding, hematoma, heparin-induced thrombocytopenia, thrombosis, or mechanical events) were checked within daily progress records.

### 2.7. Outcome

The primary outcome of this study was to assess advantages of ECCO2R therapy over conventional treatment alone in terms of 28-day ICU survival, iMV duration, and LOS.

### 2.8. Statistical analyses

A power analysis was performed with a free software (G*Powerv: 3.1.9.4, Germany) before the data collection, that at least 44 ECOO2R patients were required in order to gain an approximated power of 80% with 0.5 effect size, 0.05 alpha error probability, and 2.0 critical t-value in two tailed calculations. Collected data management and analyses were performed using statistical software program SPSS (IBM, SPSS Statistics v: 25.0, IL, USA, 2017). Comparability of the data was provided by stratum and weighting. Continuous variables were reported as median and inter quartile range (IQR), mean ± standard deviation (SD) and categorical variables as counts and proportions when appropriate. Comparison of proportions was made using Chi-square test. Data at different times during ECCO2R (repeated measures) were compared using analysis of variance (ANOVA). When significance (p ≤ 0.05) obtained after 72 h of ECCO2R, was compared with previous using paired t test (adjusted), and by Bonferroni corrections. The conditional analyses were used rather than unconditional since strata was relatively small. The contingency tables were formed. Relative and absolute risk reductions ((RRR and ARR), number need to treat (NNT), and odds ratios (OR) were calculated. All P values were two-tailed and values < 0.05 (CI of 95%) were deemed as significant.

## 3. Results

Medical records of 1715 patients were scanned. After exclusion of 164 patients with both COPD and ARDS diagnoses, 395 patients who required iMV support due to COPD (n = 256) or ARDS (n = 139) were assigned into the study. The main reason for COPD admissions was acute and severe exacerbation of disease (94%). ARDS was due to primary pulmonary insults in 74% of the cases.

General characteristics, admission SOFA scores, ABG and ventilatory parameters, iMV support duration, LOS and 28-day survival status of patients were presented in Table 1. ECCO2R therapy was noted in 75 of 395 patients. ECCO2R group 28-day survival rate was 68% and significantly higher than 58% survival rate of controls (p = 0.025). In subgroup analyses, survival rates of COPD (65%) and ARDS (73%) patient who received ECCO2R therapy were higher than control COPD (55%) and ARDS (65%) patients. In addition to this, ECCO2R therapy significantly shortened iMV duration and ICU LOS in both COPD and ARDS patients. Calculated total survival OR was found below 2.0 (1.5 (0.9–2.6)), but NNT (NNT = 10), relative risk reduction (0.16 (0.03–0.39), and absolute risk reduction rates (0.10 (0.02–0.21)) were promising for ECCO2R therapy group, as presented in Table 2. 

**Table 1:** Presentation of COPD and ARDS patients’ general characteristics, SOFA scores, arterial blood gas and ventilatory parameters, iMV days, LOS and 28-day survival rates. Frequency, percentage, median - IQR, and mean ± SD values were used as appropriate.

	TOTAL (n = 395)			COPD (n = 256)			ARDS (n = 139)		
	ECCO2R(n = 75)	Control(n = 320)	p	ECCO2R(n = 49)	Control(n = 207)	p	ECCO2R(n = 26)	Control(n = 113)	p
n, male/female	41/34	186/134		22/27	93/114		19/7	93/20	
Age (years)	63 (17 - 93)	66 (16–97)	ns	68 (51–93)	69 (41–97)	ns	58 (17–78)	64 (16–94)	ns
SOFA score	13 (8 - 17)	12 (7–16)	ns	14 (8–17)	11 (7–16)	ns	14 (11–16)	14 (9–16)	ns
ABG									
pH	7.156 ± 0.07	7.184 ± 0.02	ns	7.204 ± 0.05	7.235 ± 0.06	ns	7.133 ± 0.11	7.144 ± 0.18	ns
PaCO2 (mmHg)	81 ± 21	77 ± 19	ns	78 ± 21	75 ± 18	ns	86 ± 21	79 ± 18	ns
PaO2 (mmHg)	61 ± 6	59 ± 7	ns	80 ± 6	78 ± 7	ns	56 ± 5	55 ± 7	ns
Ventilatory									
P/F ratio	89 ± 11	92 ± 12	ns	129 ±13	133 ± 14	ns	76 ±8	81 ± 9	ns
PEEP (cmH2O)	13 ± 3	12 ± 3	ns	13 ±3	12 ± 4	ns	15 ±4	13 ± 5	ns
Pplat (cmH2O)	36 ± 6	34 ± 7	ns	35 ±6	33 ± 5	ns	38 ±7	35 ± 6	ns
ΔP (cmH2O)	23 ± 2	22 ± 3	ns	22 ±2	21 ± 2	ns	23 ±4	22 ± 2	ns
Tv/PBW (mL/kg)	7 ± 1	7 ± 2	ns	7 ±1	7 ± 1	ns	6 ±2	7 ± 2	ns
Outcomes									
iMV (days)	11 (8–19)	14 (7–22)	0.007	11 (7–18)	15 (8–21)	0.001	10 (8–17)	15 (9–25)	0.012
LOS (days)	17 (7–28)	26 (5–45)	0.032	17 (7–19)	21 (5–37)	0.035	15 (7–23)	29 (13–52)	0.025
n, survived/ex (%)	51 / 24 (68%)	187/133 (58%)	0.025	32/17 (65%)	11 /94 (55%)	0.036	19/7 (73%)	74/39 (65%)	0.042

COPD; chronic obstructive pulmonary disease, ARDS; acute respiratory distress syndrome, SOFA score; sequential organ failure assessment score, iMV; invasive mechanic-ventilation duration, LOS; length of ICU stays, IQR; inter-quartile range, ABG; arterial blood gas, P/F; PaO2/FiO2 ratio, PEEP; positive end expiratory pressure, Pplat; plateau pressure, ΔP; (driving pressure) = Pplat-PEEP, Tv/BPW; tidal volume/predicted body weight, ECCO2R; extracorporeal CO2 removal.

**Table 2:** The 2x2 contingency table presenting 28-day survival statuses of ECCO2R vs. conventional treated-alone COPD and ARDS patients, comparisons, and statistical calculations. Statistical p values, RRR, ARR, and ORs with 95% CIs.

		Survived (n)	Ex (n)	p	RRR	ARR	OR	NNT
TOTAL	ECCO2R	51 (68%)	24 (32%)	0.025	0.16(0.03–0.39)	0.10(0.02–0.21)	1.5(0.9–2.6)	10
	Control	187 (58%)	133 (42%)					
COPD	ECCO2R	32 (65%)	17 (35%)	0.036	0.20(0.06–0.52)	0.11(0.04–0.25)	1.6(0.8–3.0)	9
	Control	113 (55%)	94 (45%)					
ARDS	ECCO2R	19 (73%)	7 (27%)	0.042	0.12(0.02–0.46)	0.08(0.03–0.12)	1.4(0.6–3.7)	13
	Control	74 (65%)	39 (35%)					

ECCO2R; extracorporeal CO2 removal, COPD; chronic obstructive pulmonary disease, ARDS; acute respiratory distress syndrome, RRR; relative risk reduction, ARR; absolute risk reduction, NNT; number need to treat, OR; odds ratio, CI; confidence intervals.

Arterial blood gas, ventilatory, clinical, and laboratory parameters recorded at t = 0 and t = 72 of ECCO2R procedure, and ECCO2R duration (days), pump flow and sweep gas flow rates were presented in Table 3. The pH, PaO_2_, and PaCO_2_ levels of the control group were not significantly improved at t = 72. On the other hand, ECCO2R therapy significantly ameliorated patients ABG parameters. Significant improvement in mean pH (p = 0.048) and PaO_2_ (p = 0.032), and significant reduction in mean PaCO_2_ (p = 0.027) levels were provided at t = 72 of ECCO2R treatment. While ventilatory parameters were improved and facilitated LPV, the mean P/F ratio (p = 0.008), Pplat (p = 0.035) and driving pressure (p = 0.040) were significantly improved in both COPD and ARDS patients. A significant change in Tv/PBW levels were not detected that were about 7 mL/kg throughout 72 h. Mean PEEP levels were improved in COPD (p = 0.040) patients. While in ARDS patients, required PEEP levels were not changed in 72 h. possible due to prolonged need for higher PEEP levels in those patients.

**Table 3:** ECCO2R procedure recordings at t = 0 and t = 72 h of treatment; arterial blood gas, ventilatory, clinical and laboratory parameter comparisons. Control group pH, PaCO2, and PaO2

	TOTAL (n = 75)			COPD (n = 49)			ARDS (n = 26)		
	t = 0	t = 72	p	t = 0	t = 72	p	t = 0	t = 72	p
ABG									
Case-pH	7.156 ± 0.07	7.339 ± 0.05	0.048	7.204 ± 0.05	7.355 ± 0.06	0.050	7.133±0.11	7.332 ± 0.05	0.045
Control-pH	7.184 ± 0.02	7.265 ± 0.09	ns	7.235 ± 0.06	7.289 ± 0.09	ns	7.133 ±0.11	7.246 ± 0.11	ns
Case-PaCO2 (mmHg)	81 ± 21	53 ± 9	0.027	78 ± 21	54 ± 10	0.030	86 ±21	52 ± 8	0.020
Control-PaCO2 (mmHg)	77 ± 19	71 ± 16	ns	75 ± 18	70 ± 17	ns	79 ±18	73 ± 14	ns
Case-PaO2 (mmHg)	61 ± 6	82 ± 8	0.032	80 ± 6	82 ± 8	0.040	56 ±5	83 ± 8	0.020
Control-PaO2 (mmHg)	59 ± 7	71 ± 9	ns	78 ± 7	80 ± 6	ns	55 ±7	61 ± 6	ns
Ventilatory									
P/F ratio	89 ± 11	150 ± 53	0.008	129 ± 13	166 ± 52	0.020	76 ± 8	142 ± 54	0.001
PEEP (cmH2O)	13 ± 3	12 ± 3	0.050	13 ± 3	10 ± 2	0.040	15 ± 4	16 ± 3	ns
Pplat (cmH2O)	36 ± 6	29 ± 3	0.035	35 ± 6	28 ± 4	0.030	38 ± 7	32 ± 2	0.050
ΔP (cmH2O)	23 ± 2	17 ± 3	0.040	22 ± 2	18 ± 3	0.040	23 ± 4	16 ± 3	0.040
Tv/PBW (mL/kg)	7 ±1	7 ± 1	ns	7 ± 1	7 ± 2	ns	6 ± 2	6 ± 1	ns
Clinical									
RR (/min)	19 ± 2	18 ± 3	ns	16 ± 2	15 ± 3	ns	24 ± 3	25 ± 2	ns
HR (/min)	117 ± 13	113 ±13	ns	112 ± 15	108 ± 14	ns	127 ± 9	122 ± 11	ns
MAP (mmHg)	58 ± 9	54 ± 7	ns	58 ± 12	54 ± 8	ns	56 ± 6	52 ± 4	ns
Laboratory									
Hemoglobin (g/dL)	11.3 ± 0.8	10.5 ± 0.8	0.050	11.6 ± 0.8	10.8 ± 0.6	0.050	10.7 ± 0.8	9.9 ± 1.1	0.050
PLT (10^3/µ)	117 ± 17	103 ± 18	0.043	121 ± 14	107 ± 19	0.040	109 ± 21	95 ± 17	0.050
PT-INR	1.3 ± 0.2	1.4 ± 0.2	ns	1.3 ± 0.2	1.4 ± 0.2	ns	1.1 ± 0.2	1.2 ± 0.2	ns
aPTT ratio (s)	37 ± 8	73 ± 15	0.023	34 ± 8	68 ± 14	0.020	42 ± 9	85 ± 18	0.030
ECCO2R									
ECCO2R days	6 (4–9)			7 (4–9)			6 (4–8)		
Pump flow (mL/min)	270 (190–420)			270 (180–420)			320 (210–450)		
Sweep gas flow rate (L/min)	8 (6–11)			7 (6–10)			8 (6–12)		

ECCO2R; extracorporeal CO2 removal, IQR; inter-quartile range, COPD; chronic obstructive pulmonary disease, ARDS; acute respiratory distress syndrome, ABG; arterial blood gas, P/F; PaO2/FiO2 ratio, PEEP; positive end expiratory pressure, Pplat; plateau pressure, ΔP; (driving pressure) = Pplat - PEEP, Tv/BPW; tidal volume/predicted body weight, RR; respiratory rate, HR; heart rate, MAP; mean arterial pressure, ns; nonsignificant.

There were no detected significant change or deterioration in patients’ mean RR, HR, and MAP parameters through 72 h. of ECCO2R therapy. The mean hemoglobin and PLT levels were found lower at t = 72 but those were not clinically remarkable nor required transfusion, as in daily progress records. The aPPT ratio was 2× longer due to provided heparin anti-coagulation protocol, at t = 72. No severe adverse effects related to procedure were mentioned in the records.

At the final step, we re-tested the hypothesis with a strict 1:1 matching by SOFA disease severity score. While 44 patients from ECCO2R and 75 patients from control group were excluded by additionally re-defined criteria, downsized the case and control samples to 31/31 (COPD n = 20/20, ARDS n = 11/11). Figure 2. Total survival rates were in favor of ECCO2R patients (68%) against controls (56%). In subgroup comparisons, survival rates of ECCO2R treated COPD (75%) and ARDS (55%) patients were significantly higher than control COPD (50%) and ARDS (18%). The contingency calculations for matched comparisons were presented in Table 4. Although resulted sample size was highly downsized by this strict matching, total survival OR (1.7 (0.8–3.7)) and NNT (NNT = 8) values strikingly favored beneficial effect of ECCO2R therapy.

**Table 4:** The 2x2 contingency table presenting 28-day survival statuses of ECCO2R vs. conventional treated-alone COPD and ARDS patients, for 1:1 matching. Statistical p values, RRR, ARR, and ORs with 95% CIs.

		Survived (n)	Ex (n)	p	RRR	ARR	OR	NNT
TOTAL	ECCO2R	21 (68%)	10 (32%)	0.018	0.22(0.07–0.60)	0.12(0.05–0.30)	1.7(0.8–3.7)	8
	Control	136 (56%)	109 (44%)					
COPD	ECCO2R	15 (75%)	5 (25%)	0.016	0.50(0.10–1.49)	0.25(0.04–0.54)	3.0(0.8–11.4)	4
	Control	10 (50%)	10 (50%)					
ARDS	ECCO2R	6 (55%)	5 (45%)	0.021	2.0(0.2–10.7)	0.36(0.09–0.74)	5.4(0.8–37.5)	3
	Control	2 (18%)	9 (82%)					

ECCO2R; extracorporeal CO2 removal, COPD; chronic obstructive pulmonary disease, ARDS; acute respiratory distress syndrome, RRR; relative risk reduction, ARR; absolute risk reduction, NNT; number need to treat, OR; odds ratio, CI; confidence intervals.

## 4. Discussion

Recently, ECCO2R therapy has been more frequently used in treatment of patients with severe respiratory failure. Especially in conditions such as ARDS and acute exacerbations of COPD that LPV is required and lifesaving. ECCO2R facilitates settings of lower respiratory rates, and lower driving and plateau pressures, while successfully removing excess CO_2_. Additionally, removal of excess CO_2_ also would help to normalize acidotic pH levels and improve manageability of distressed conditions.

However, in a previous systematic review that included two RCT and 12 observational studies on ARDS patients (n = 495), ECCO2R therapy was not found advantageous in terms of patient survival and LOS [23]. On the contrary, our study results showed a significant survival and LOS benefit with ECCO2R therapy. We assumed that difference was related to mixed type of diagnoses in our study, in which ARDS patients constituted the one third of the sample. It was also reasonable to expect lower survival rates in ARDS patients than COPD. Nevertheless, subgroup analyses of our ARDS patients presented higher survival benefit with ECCO2R. In addition, iMV duration and ICU LOS were significantly shorter. Then, a supportive systematic review to our assumptions, in 2015 by Sklar et al., that included ten case series of ECCO2R therapy in COPD patients with hypercapnic respiratory failure (n = 87), showed that ECCO2R therapy assisted successful extubation (53%) with lower mortality rates, and improved ABG parameters [24]. The patient characteristics of Sklar’s sample were more similar to our study patients, hence we assume those results were more comparable than previous ones.

Eventually, the ECLAIR study in 2016 was published that ECCO2R therapy was successful to avoid iMV and shortened iMV duration, but not LOS nor improved survival [15]. This was a multicentric case-control (n = 25/25) study that compared hypercapnic respiratory failure patients, and especially focused on avoidance of intubation and iMV by ECCO2R, rather than survival benefits. These results were valuable but should be cautiously evaluated in regards of small sample size for generalizability. Another feasibility study on ECCO2R therapy in ARDS patients (n = 15) by Fanelli et al., reported significant improvements in clinical, ventilator, and ABG parameters similar to our results. They underlined the efficiency of ECCO2R in providing LPV, that was accounted as one of the most effective approaches in ARDS patient [16]. Fanelli’s study ARDS sample was also quite similar to our ARDS patients in terms of age, sex, and SOFA scores, and were consistent with our results.

Here, we also presented that 72 h of ECCO2R procedure significantly improved ABG and ventilatory parameters in both COPD and ARDS patients and safely provided LPV settings. Thereby, we assumed this was the main influential factor for higher survival rates. Another supportive study by Hilty et al. assessed 20 ARDS and COPD patients with hypercapnic respiratory failure, although a conventional controls arm was not present, also reported that ECCO2R therapy was safe and provided LPV [25]. Another similar retrospective observational ECCO2R study by Moss et al. that included 14 patients (COPD n = 5, ARDS n = 9) with hypercapnic respiratory failure, compared the data of survivors vs. nonsurvivors [26]. The mean pH levels were successfully improved, and the survival rate was 71% (10/14), in concordance with our results. However, the average LOS (31.9 vs*.* 7) and iMV (53 vs*.* 8.5) were longer in survivors than nonsurvivors. That discrepancy could be due to lack of a conventional treatment control group for a coherent comparison in that study.

However, in 2017, Taccone et al. reviewed six studies on ECCO2R therapy in COPD and ARDS patients (n = 142) those were published between 1994–2015, and concluded that evidence of survival benefit was moderate, yet [7]. Although inconclusive data have been published until 2018, we think these should be considered at respect of that most studies were case series, not homogenous with possible case-mix bias issues and lack of control groups. As of 2018, more promising results have given to rise. A UK Register study on severe respiratory failure patients (n = 60) reported significant benefits of ECCO2R treatment on ABG and ventilatory parameters, without any clinical deterioration, however not showed an exact benefit for survival [27]. Proceeded by Schmidt et al., 20 ARDS patients treated by ECCO2R and safely enhanced LPV with significantly higher survival rates (85%) [14]. Finally, the recent SUPERNOVA study on feasibility of ECCO2R therapy in 95 ARDS patients reported significantly high cumulative 28-day survival rates (73%) [13]. In addition, a recently published retrospective data of 11 respiratory failure patients by Grasselli et al. reported that low-flow VV-ECCO2R successfully improved ABG parameters and reduced ventilatory load, with a 71% survival rate in COPD patients [28].

The results of our study have supported and in concordance with accumulated data as of 2018, but not previous ones [13,14,28]. A positive attitude has been rising on extra-corporeal therapies by 2018. Technological advancements, procedural improvements, and easier accessibility of ECCO2R should has contributed to this trend. Promising benefits of ECCO2R has gained a wider recognition and concern. Recently, the first European consensus encouraging ECCO2R therapy in ICU has been published by Combes et al., based on accumulated data that ARDS and COPD patients could benefit from ECCO2R [18]. As soon, we could anticipate that ECCO2R would be a part of conventional treatment protocols in severe ARDS and COPD patients.

Our study has number of limitations to be considered. The retrospective design was prone to recall and selection biases. The main analyses were depended on case-mix samples. The secondary analyses with strict matching provided a specific comparability but led high number of exclusions and downsized the sample. These could have produced exaggerated statistical significances. Finally, this study represents to our patient population and applicable to our settings, reasonably cannot be extrapolated to all settings and external validations are required for decisive evidence.

## 5. Conclusion

Results of our study supported that low-flow VV-ECCO2R therapy has a role in improving survival rates, iMV duration, and LOS in patients with HRF due to COPD or ARDS. In addition, significant improvements in ABG and ventilation parameters while facilitating LPV have been achieved by ECCO2R therapy, with no marked clinical and laboratory deteriorations. Further studies are required to assess that promising benefits of ECCO2R therapy.

## Informed consent

This study protocol received institutional review board approval from Bioethical Board of Trakya University (no= 2020/199–09/ 05), and that all participants or (in case) legally authorized surrogates provided informed consent in the format required by the relevant authorities.
